# The Effect of *Limosilactobacillus reuteri* on Social Behavior Is Independent of the Adaptive Immune System

**DOI:** 10.1128/msystems.00358-22

**Published:** 2022-10-26

**Authors:** Sean W. Dooling, Martina Sgritta, I-Ching Wang, Ana Luiza Rocha Faria Duque, Mauro Costa-Mattioli

**Affiliations:** a Department of Neuroscience, Baylor College of Medicine, Houston, Texas, USA; b Memory and Brain Research Center, Baylor College of Medicine, Houston, Texas, USA; c Department of Molecular and Human Genetics, Baylor College of Medicine, Houston, Texas, USA; d Department of Food and Nutrition, School of Pharmaceutical Sciences, São Paulo State University (UNESP), Araraquara, Brazil; Istanbul Medipol University School of Medicine

**Keywords:** *Limosilactobacillus reuteri*, oxytocin, social behavior, adaptive immune system, gut-microbiota-brain axis, *Lactobacillus reuteri*

## Abstract

Gut microbes can modulate almost all aspects of host physiology throughout life. As a result, specific microbial interventions are attracting considerable attention as potential therapeutic strategies for treating a variety of conditions. Nonetheless, little is known about the mechanisms through which many of these microbes work. Recently, we and others have found that the commensal bacterium Limosilactobacillus reuteri (formerly Lactobacillus reuteri) reverses social deficits in several mouse models (genetic, environmental, and idiopathic) for neurodevelopmental disorders in a vagus nerve-, oxytocin-, and biopterin-dependent manner. Given that gut microbes can signal to the brain through the immune system and L. reuteri promotes wound healing via the adaptive immune response, we sought to determine whether the prosocial effect mediated by L. reuteri also depends on adaptive immunity. Here, we found that the effects of L. reuteri on social behavior and related changes in synaptic function are independent of the mature adaptive immune system. Interestingly, these findings indicate that the same microbe (L. reuteri) can affect different host phenotypes through distinct mechanisms.

**IMPORTANCE** Because preclinical animal studies support the idea that gut microbes could represent novel therapeutics for brain disorders, it is essential to fully understand the mechanisms by which gut microbes affect their host’s physiology. Previously, we discovered that treatment with Limosilactobacillus reuteri selectively improves social behavior in different mouse models for autism spectrum disorder through the vagus nerve, oxytocin reward signaling in the brain, and biopterin metabolites (BH4) in the gut. However, given that (i) the immune system remains a key pathway for host-microbe interactions and that (ii) L. reuteri has been shown to facilitate wound healing through the adaptive immune system, we examined here whether the prosocial effects of L. reuteri require immune signaling. Unexpectedly, we found that the mature adaptive immune system (i.e., conventional B and T cells) is not required for L. reuteri to reverse social deficits and related changes in synaptic function. Overall, these findings add new insight into the mechanism through which L. reuteri modulates brain function and behavior. More importantly, they highlight that a given bacterial species can modulate different phenotypes (e.g., wound healing versus social behavior) through separate mechanisms.

## INTRODUCTION

Gut microbes are fundamental to nearly every aspect of host physiology and fitness (1, 2), including brain function and behavior. Indeed, a large body of preclinical literature has uncovered a bidirectional communication system linking the gut and the brain, known as the gut-microbiota-brain axis (3–5). Briefly, foundational studies have demonstrated that germ-free mice and mice treated with broad-spectrum antibiotics exhibit behavioral abnormalities, including endophenotypes associated with neurological disorders, such as autism spectrum disorder (ASD) (6–11). In addition, using experimental mouse models, we and others have shown that gut microbes can modulate endophenotypes for complex neurological disorders in a very powerful way (3, 5, 12–14). In particular, social behavior has emerged as an endophenotype that is strongly regulated by the gut-microbiota-brain axis across species (9, 13, 15–18).

Humans with specific neurological disorders, such as ASD, often possess a different gut microbiota composition (19–23) and are often afflicted with gastrointestinal (GI) symptoms. Furthermore, recent studies have shown that microbiota transfer therapy and dietary modulation of the gut microbiota alleviates both GI and behavioral symptoms in children with certain neurological dysfunction, including ASD (24–26). Thus, the gut microbiome is emerging as an important modulator of both brain development/function and complex behaviors, including social behavior.

Originally, we found that treatment with the bacterial species Limosilactobacillus reuteri (formerly Lactobacillus reuteri [27]) selectively improves social behavior, but not other behavioral abnormalities, in a maternal obesity model (i.e., maternal high-fat diet offspring) (13) for ASD. In subsequent studies, we found that L. reuteri promoted social behavior in genetic (*Shank3B^−/−^* and *Cntnap2*^−/−^), environmental (valproic acid and germ-free), and idiopathic (BTBR) mouse models of social deficits (14, 28). Importantly, and consistent with our results, two independent studies show that L. reuteri reversed the social deficits in *Shank3B^−/−^* mice (29) and BTBR mice (30). Thus, the finding that L. reuteri promotes social behavior is strongly supported by numerous, convergent discoveries in several animal models and across different levels of analysis and laboratories.

However, to fully understand how the gut microbiota modulate brain function and behavior, it is necessary to dissect the underlying molecular, cellular, and systems mechanisms. There are several possible ways by which gut microbes can influence the brain. These include (i) vagus nerve signaling (31), (ii) circulation of microbial metabolites through the blood (32, 33), and (iii) modulation of the immune system (34, 35). Our initial mechanistic studies revealed that L. reuteri acts in a vagus nerve-dependent manner and rescues social interaction and social interaction-induced synaptic plasticity in the ventral tegmental area (VTA) of ASD mice, but not in oxytocin receptor-deficient mice (14). More recently, we found that L. reuteri acts by promoting biopterin metabolite (BH4) levels in the host’s gut (28).

A previous study showed that L. reuteri facilitates wound healing through the stimulating a subpopulation of T cells as a part of the adaptive immune response (36), a process also involving signaling through the vagus nerve and oxytocin (36). While there appears to be much overlap in the mechanisms through which L. reuteri improves social behavior and wound healing (i.e., vagus nerve and oxytocin signaling), it is currently unknown whether the adaptive immune system plays a role in the effects of L. reuteri on social behavior. Therefore, we sought to test the hypothesis that the adaptive immune system, namely, conventional adaptive B and T lymphocytes, also mediates the L. reuteri prosocial effect.

Using genetic, behavioral, molecular, and electrophysiological approaches in this study, we found that L. reuteri reverses the social deficits in a genetic mouse model for ASD lacking mature conventional B and T cells. Accordingly, L. reuteri reverses deficits in oxytocin and synaptic potentiation associated with social reward. Thus, the adaptive immune system is not a major contributor to the L. reuteri-mediated rescue of social behavior. Consequently, the mechanisms through which a given bacterial species modulates different aspects of host physiology are not fully shared.

## RESULTS

### *Shank3B^–/–^* mice exhibit no deficits in adaptive immune system maturation and *L. reuteri* treatment does not affect mature lymphocyte levels.

The gut microbiota play an important role in the host’s immune signaling (37). For example, gut microbes can modulate immune responses by triggering both proinflammatory (38) and anti-inflammatory responses (39, 40). Interestingly, aberrant immune responses may contribute to some aspects of ASD symptomatology (41), which often features with gastrointestinal comorbidities and alterations in the gut microbiome (42–44).

Specifically, L. reuteri has been shown to modulate the adaptive immune system (45–48) and facilitate wound healing (36). However, whether the effect of L. reuteri on social behavior also requires the adaptive immune response remains unknown. To answer this question, we used *Shank3B^−/−^* mouse model for neurodevelopmental disorders because (i) they exhibit social deficits that are reproduced across laboratories (14, 29, 49); (ii) they have a deficient oxytocinergic system (14), which is known to modulate social behaviors and is implicated in ASD (50, 51); and (iii) we and others have previously shown that L. reuteri reverses their social deficits (14, 29).

**FIG 1:**
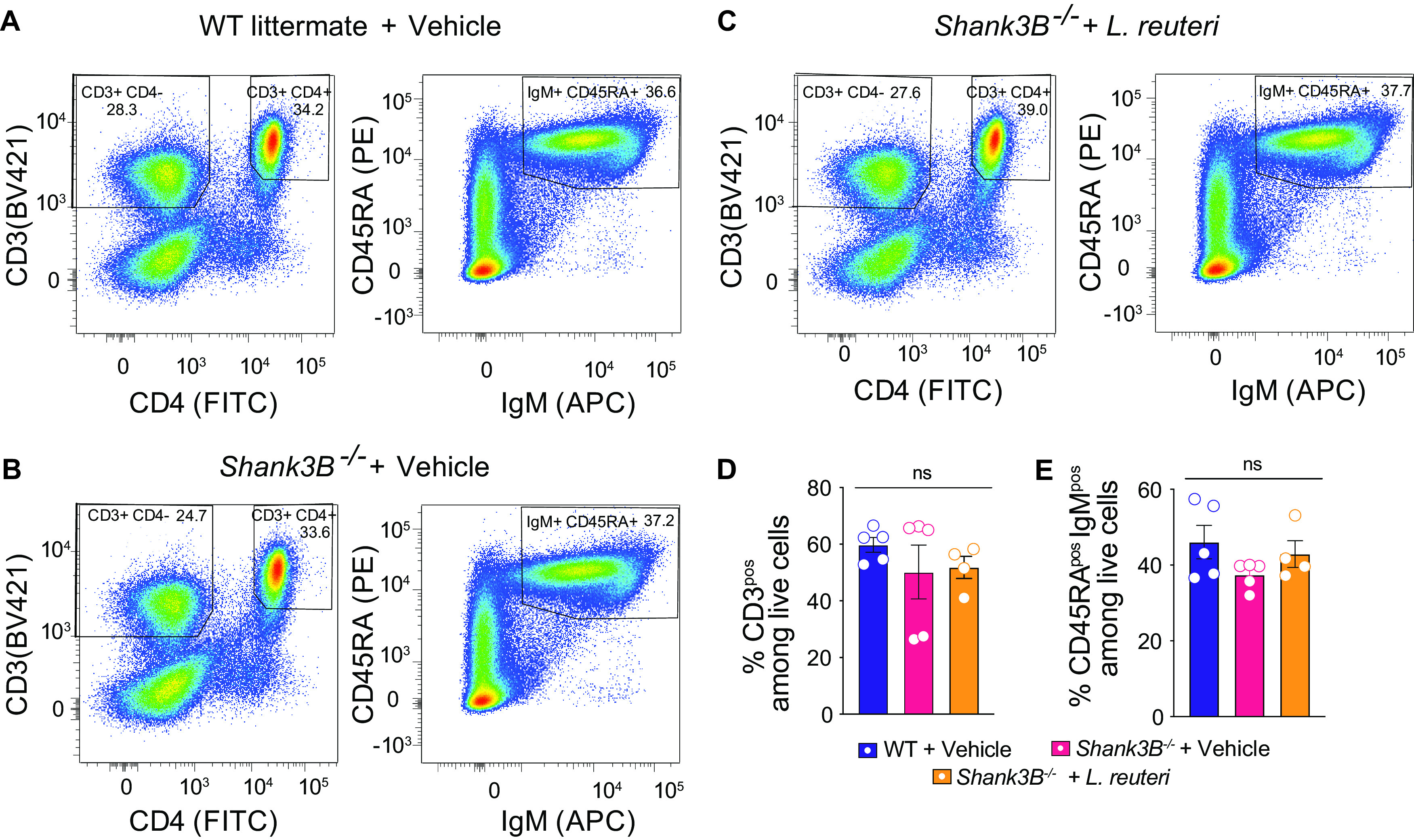
*Shank3B^−/−^* mice do not show changes in the percentages of mature T and B lymphocytes compared to their controls, and L. reuteri does not influence the number of adaptive immune cells. (A to D) Flow cytometry analysis of CD3, CD4, IgM, and CD43RA expression in the spleen cells of WT littermate + vehicle, *Shank3B^−/−^* + vehicle, and *Shank3B^−/−^ +* L. reuteri (*n* = 4 to 5 mice per group). (A) Distribution of CD3, CD4, IgM, and CD43RA signal in WT littermate + vehicle. (B) Distribution of CD3, CD4, IgM, and CD43RA signal in *Shank3B^−/−^* + vehicle. (C) Distribution of CD3, CD4, IgM, and CD43RA signal in *Shank3B^−/−^* + L. reuteri. (D) Percentage of all CD3^+^ cells. (WT littermate versus *Shank3B^−/−^*: q = 2.646, *P* = 0.1929; *Shank3B^−/−^* versus *Shank3B^−/−^ +* L. reuteri: q = 1.576, *P* = 0.5252; WT littermate versus *Shank3B^−/−^ +* L. reuteri: q = 0.9181, *P* = 0.7966 [one-way ANOVA with Tukey *post hoc* test, *P* = 0.2131]). (E) Percentage of IgM^+^ and CD43RA^+^ cells. (WT littermate versus *Shank3B^−/−^*: q = 1.544, *P* = 0.5382; *Shank3B^−/−^* versus *Shank3B^−/−^ +* L. reuteri: q = 0.2462, *P* = 0.9835; WT littermate versus *Shank3B^−/−^ +* L. reuteri: q = 1.210, *P* = 0.6779 [one-way ANOVA with Tukey *post hoc* test, *P* = 0.5310]). ns, nonsignificant. Bar graphs show means ± the SEM with individual data points.

We first examined whether the maturation of the adaptive immune system is altered in *Shank3B^−/−^* mice compared to control littermates. To this end, we measured the number of T (CD3^+^ CD4^+^ and CD3^+^ CD4^–^) and B (IgM^+^ and CD43RA^+^) lymphocytes in control mice, *Shank3B*^−/−^ mice treated with vehicle, and *Shank3B^−/−^* mice treated with L. reuteri using flow cytometry (Fig. 1A to C). We found that there was no significant difference in the percentages of mature T and B lymphocytes between WT control and *Shank3B^−/−^* mice (Fig. 1D and E). Moreover, L. reuteri treatment did not alter the percentages of mature T and B cells in *Shank3B^−/−^* mice (Fig. 1D and E). Hence, *Shank3B^−/−^* mice do not exhibit deficits in adaptive immune system maturity, and L. reuteri treatment does not affect the overall number of the mature adaptive immune system cells in *Shank3B^−/−^* mice.

### *L. reuteri* treatment corrects social deficits in *Shank3B^–/–^* mice lacking a mature adaptive immune system.

**FIG 2:**
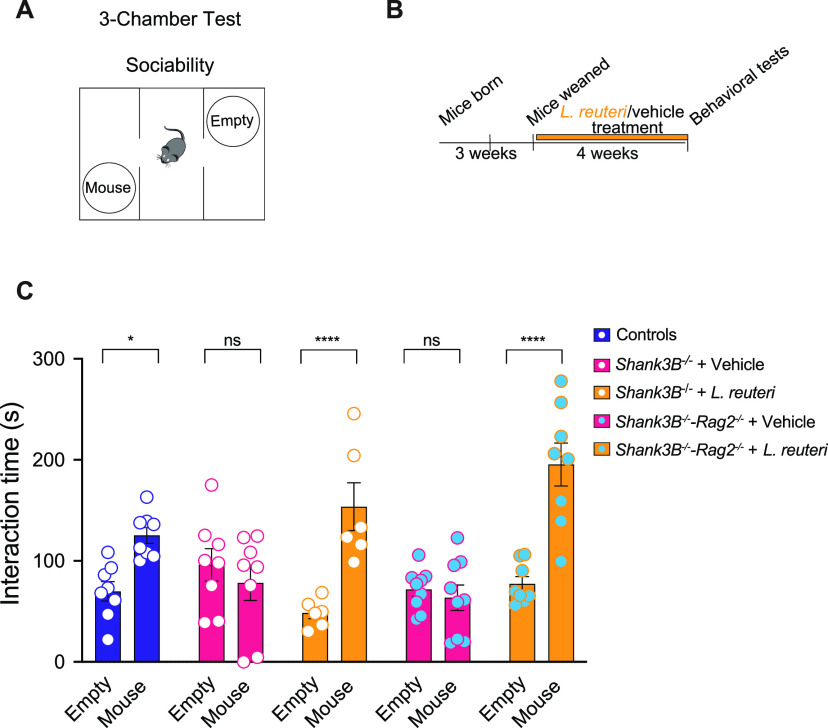
L. reuteri corrects social deficits in *Shank3B^−/−^-Rag2^−/−^* mice. (A) Schematic of the three-chamber social behavior test. (B) Schematic of the experimental design. (C) Social interaction in vehicle- and L. reuteri-treated WT, *Shank3B^−/−^*, and *Shank3B^−/−^-Rag2^−/−^* mice in the three-chamber test (*n* = 8 to 9 per group; WT littermate: *t* = 2.929, *P* = 0.0194; *Shank3B^−/−^*: *t* = 0.9491, *P* > 0.9999; *Shank3B^−/−^* + L. reuteri: *t* = 4.738, *P* < 0.0001; *Shank3B^−/−^-Rag2^−/−^* + vehicle: *t* = 0.4571, *P* > 0.9999; *Shank3B^−/−^-Rag2^−/−^* + L. reuteri: *t* = 6.247, *P* < 0.0001 [two-way ANOVA with Bonferroni correction, F_(4,68)_ = 10.34; *P* < 0.0001]). *, *P* < 0.05; ****, *P* < 0.0001; ns, nonsignificant. Bar graphs show means ± the SEM with individual data points.

The deletion of *Rag2* disrupts the formation of the Rag complex and halts B cell and T cell development at the pro-B and the pro-T cell stages prior to V(D)J recombination of B cell and T cell receptors, thus preventing them from reaching full maturation (52). Consequently, *Rag2^−/−^* mice are a widely used animal model to study deficiencies in adaptive immunity (53–56). We first sought to determine whether genetic ablation of the mature adaptive immune system via the deletion of *Rag2* would alter social behavior. To test social behavior, we performed a three-chamber test for sociability. In this behavioral task (Fig S1A, Fig. 2A), mice can choose either a nonsocial interaction, with an empty cup (Empty), or a social interaction, with an unfamiliar mouse (Mouse). As expected, control (wild-type [WT]) mice display normal social interaction as they spend more time interacting with the mouse instead of the empty cup (Fig S1B). *Rag2^−/−^* mice also interacted more with the mouse than the cup, indicating that the loss of the Rag2-recombined adaptive immune system does not lead to impaired social behavior.

10.1128/msystems.00358-22.1 FIG S1 *Rag2^−/−^* mice display normal social behavior. (A) Schematic of the three-chamber social behavior test. (B) Social interaction in WT and *Rag2^−/−^* mice in the three-chamber test (*n* = 12 to 13 per group; WT: *t* = 7.518, *P* < 0.0001; *Rag2^−/−^*: *t* = 7.302, *P* < 0.0001 [two-way ANOVA with Bonferroni correction, F_1,46_ = 0.003248, *P* = 0.9548]). Download FIG S1, EPS file, 1.9 MB. Copyright © 2022 Dooling et al. 2022 Dooling et al. https://creativecommons.org/licenses/by/4.0/ This content is distributed under the terms of the Creative Commons Attribution 4.0 International license.

To test whether L. reuteri rescues social behavior in the *Shank3B^−/−^* mice through modulation of mature B and T cells, we crossed *Shank3B*^−/−^ mice to *Rag2*^−/−^ mice to generate *Shank3B^−/−^* mice lacking mature B and T lymphocytes, here defined as “*Shank3B^−/−^-Rag2^−/−^*” mice. As expected, *Shank3B^−/−^-Rag2^−/−^* mice lack CD3^+^, CD4^+^, IgM^+^, and CD43RA^+^ cells in the spleen, indicating the absence of a mature B and T cells in the mutant mice (see Fig. S2A to D).

10.1128/msystems.00358-22.2 FIG S2 *Shank3B^−/−^-Rag2^−/−^* mice lack mature lymphocytes T and B, unlike their controls. (A and B) Flow cytometry analysis of CD3, CD4, IgM, and CD43RA expression in the spleen cells in WT *Shank3B^−/−^-Rag2^−/−^* littermates (*n* = 3 mice per group). (A) Distribution of CD3, CD4, IgM and CD43RA signal in WT littermate. (B) Distribution of CD3, CD4, IgM, and CD43RA signal in *Shank3B^−/−^-Rag2^−/−^* mice. (C) Percentage of CD3^+^ cells in WT *Shank3B^−/−^-Rag2^−/−^* littermates (unpaired *t* test: *t *=* *17.98, *P* < 0.0001). (D) Percentage of IgM^+^ and CD43RA^+^ cells in WT and *Shank3B^−/−^-Rag2^−/−^* littermates (unpaired *t* test: *t *=* *19.16, *P* < 0.0001). ****, *P* < 0.0001. Bar graphs show means ± the SEM with individual data points. Download FIG S2, EPS file, 2.6 MB. Copyright © 2022 Dooling et al. 2022 Dooling et al. https://creativecommons.org/licenses/by/4.0/ This content is distributed under the terms of the Creative Commons Attribution 4.0 International license.

*Shank3B^−/−^* mice treated with vehicle display social deficits (Fig. 2C), consistent with previous studies (14, 29, 49). Similarly, vehicle-treated *Shank3B^−/−^-Rag2^−/−^* mice also exhibited social deficits (Fig. 2C). If the prosocial effect of L. reuteri depends on the adaptive immune system, then L. reuteri would fail to rescue the social deficits in *Shank3B^−/−^*-*Rag2*^−/−^ mice. Interestingly, however, we found that L. reuteri was able to reverse the social deficits in *Shank3B^−/−^* mice with a deficient adaptive immune system (*Shank3B^−/−^-Rag2^−/−^* mice) (Fig. 2C). These data demonstrate that L. reuteri rescues social behavior independently of the adaptive immune system.

### *L. reuteri* treatment increases oxytocin levels in the paraventricular nucleus of *Shank3B^–/–^* mice lacking a mature adaptive immune system.

Oxytocin (Oxt) is an evolutionarily conserved neuropeptide critically implicated in social behavior (57). Accordingly, several animal models with abnormal social behavior, including *Shank3B*-deficient animals, feature decreased Oxt levels and Oxt treatment rescues select deficits in behavior and brain development in these mice (13, 14, 28, 58–62). In addition, Oxt has been shown to be intricately linked to immune system signaling (63, 64). Interestingly, the effects of L. reuteri on both social behavior and wound healing have been shown to be dependent on Oxt signaling (13, 28, 36). Indeed, L. reuteri increases Oxt levels in plasma and in the brain of several mouse models of ASD, including *Shank3B^−/−^* mice (13, 14, 28, 36).

**FIG 3:**
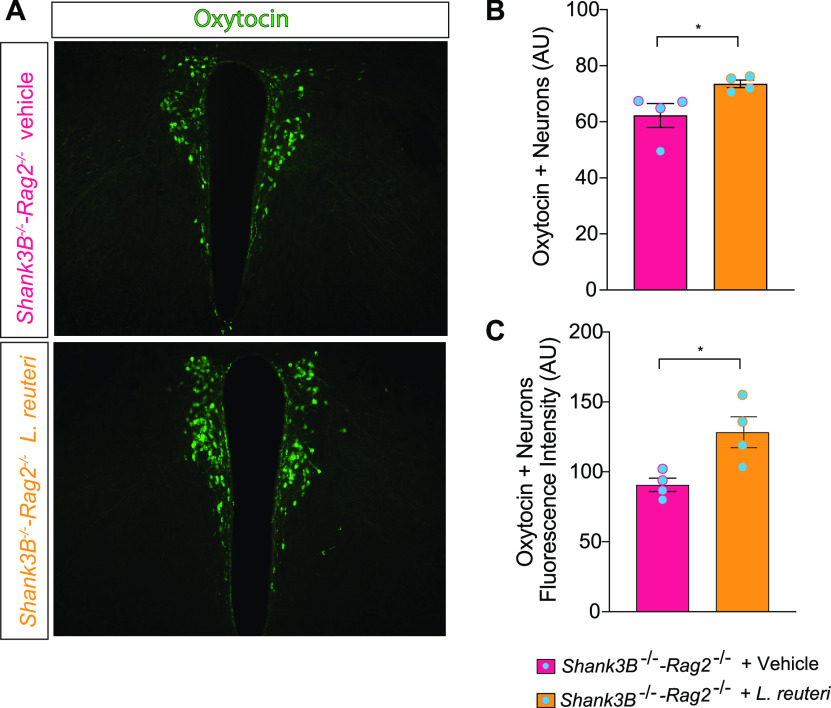
L. reuteri increases oxytocin levels in the PVN of the hypothalamus in *Shank3B^−/−^ Rag2^−/−^* mice. (A) Oxytocin immunoreactivity in the PVN of *Shank3B^−/−^-Rag2^−/−^* mice treated with either vehicle or L. reuteri. (B) Oxytocin-positive cell (*n* = 4 mice per group; *Shank3B^−/−^-Rag2^−/−^* + vehicle versus *Shank3B^−/−^-Rag2^−/−^ +* L. reuteri [unpaired *t* test, *t* = 2.519, *P* = 0.0453]). (C) Oxytocin immunofluorescence intensity (*n* = 4 mice per group; *Shank3B^−/−^-Rag2^−/−^* + vehicle versus *Shank3B^−/−^-Rag2^−/−^ +* L. reuteri [unpaired *t* test, *t* = 3.124, *P* = 0.0205]). *, *P* < 0.05. Bar graphs show means ± the SEM with individual data points.

Given that L. reuteri reversed the social deficits in *Shank3B^−/−^* mice with an impaired adaptive immune system, we next examined whether disruptions of the adaptive immune system affect L. reuteri’s ability to boost the host’s oxytocin system. To this end, we performed immunohistochemistry in paraventricular nucleus (PVN) of the hypothalamus, where Oxt is primarily produced. Consistent with the behavioral data, we found that treatment with L. reuteri was able to increase oxytocin levels in *Shank3B^−/−^-Rag2^−/−^* mice, as determined by an increased number and fluorescence intensity of Oxt-positive neurons in the mutant mice treated with L. reuteri (Fig. 3). Thus, the adaptive immune system does not affect the L. reuteri-mediated increase in Oxt.

### *L. reuteri* treatment corrects social interaction-induced synaptic potentiation in the dopaminergic neurons of the ventral tegmental area of *Shank3B^–/–^* mice lacking a mature adaptive immune system.

Brain regions that process naturally rewarding stimuli are crucially required for social behaviors (65, 66). During social interaction, oxytocin is released from neurons in the PVN and signal to oxytocin receptors on dopaminergic (DA) neurons in the ventral tegmental area (VTA), leading to the release of dopamine and facilitation of a rewarding sensation (67). We and others found that social interaction leads to an increase in synaptic potentiation in VTA DA neurons of both birds and mice (13, 68, 69). Moreover, this evolutionarily conserved process, a likely cellular model of social reward, is impaired in several mouse models for ASD (13, 14, 28). In humans with ASD, magnetic resonance imaging studies have shown deficiencies in the activity of reward regions after social behavior, further supporting the notion that reward centers of the brain are a major regulator of social behavior (70–72). More importantly, we have previously shown that L. reuteri reverses the deficits in synaptic potentiation underlying social reward in several mouse models for ASD (13, 14, 28) in an oxytocin-dependent manner (14). Moreover, we found that the L. reuteri-induced metabolite, BH4, also corrected changes in social interaction-induced synaptic potentiation (28). However, the role of the adaptive immune system has yet to be explored in this reward-related process.

**FIG 4:**
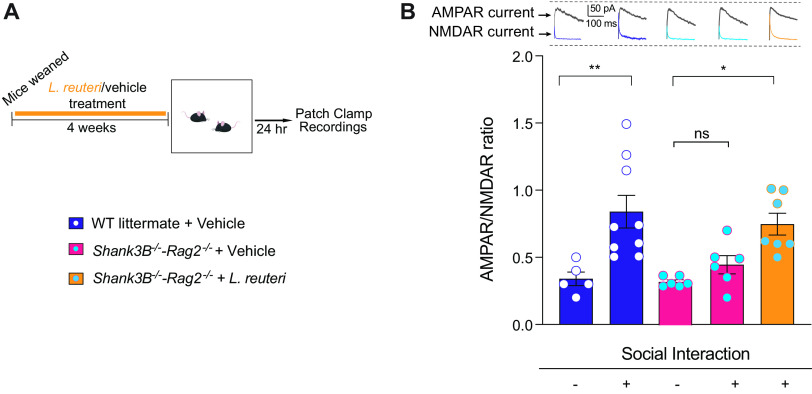
L. reuteri restores social interaction-induced synaptic transmission in the DA neurons of the lateral VTA in *Shank3B^−/−^-Rag2^−/−^* mice. (A) Schematic of the experimental design. (B) Representative traces of AMPAR and NMDAR currents (top) and AMPAR/NMDAR ratio (bottom) in dopaminergic neurons of the lateral VTA in baseline condition or following social interaction with a stranger mouse. (*n* = 5 to 9 mice per group; Control baseline versus Control + stranger interaction: q = 5.396, *P* = 0.0057; Control baseline versus *Shank3B^−/−^-Rag2^−/−^* baseline: q = 0.1656, *P* > 0.9999; Control baseline versus *Shank3B^−/−^-Rag2^−/−^* + L. reuteri *+* stranger interaction: q = 4.185, *P* = 0.0452; Control baseline versus *Shank3B^−/−^-Rag2^−/−^ +* stranger interaction: q = 1.044, *P* = 0.9457; Control + stranger interaction versus *Shank3B^−/−^-Rag2^−/−^* baseline: q = 5.900 *P* = 0.0023; Control + stranger interaction versus *Shank3B^−/−^-Rag2^−/−^* + L. reuteri *+* stranger interaction: q = 1.110, *P* = 0.9329; Control stranger interaction versus *Shank3B^−/−^-Rag2^−/−^ +* stranger interaction: q = 4.511, *P* = 0.0266; *Shank3B^−/−^-Rag2^−/−^* baseline versus *Shank3B^−/−^-Rag2^−/−^* + L. reuteri *+* stranger interaction: q = 4.585, *P* = 0.0235; *Shank3B^−/−^-Rag2^−/−^* baseline versus *Shank3B^−/−^-Rag2^−/−^ +* stranger interaction: q = 1.268, *P* = 0.8957; *Shank3B^−/−^-Rag2^−/−^* + L. reuteri *+* stranger interaction versus *Shank3B^−/−^-Rag2^−/−^ +* stranger interaction: q = 3.268, *P* = 0.1712 [one-way ANOVA with Tukey test, F_4,28_ = 7.230, *P* = 0.0004]). *, *P* < 0.05; **, *P* < 0.01; ns, not significant. Bar graphs show means ± the SEM with individual data points.

To determine whether the adaptive immune system was involved in social reward processes, we performed whole-cell patch-clamp recordings and measured the ratio of currents generated by AMPA (α-amino-3-hydroxy-5-methyl-4-isoxazolepropionic acid) and NMDA (*N*-methyl-d-aspartate) receptors (AMPAR/NMDAR ratio) in dopaminergic neurons (Fig. 4A). As expected and consistent with previous results (13, 14, 28), social interaction triggered a significant increase in the AMPAR/NMDAR ratio in WT control mice (Fig. 4B), but it failed to do so in vehicle-treated *Shank3B^−/−^*-*Rag2^−/−^* mice. The lack of a significant increase in the AMPAR/NMDAR ratio after social interaction indicates impairments in synaptic transmission associated with social reward. Interestingly, L. reuteri treatment reverses the deficits in synaptic plasticity in VTA DA neurons from *Shank3B^−/−^*-*Rag2^−/−^* mice (Fig. 4B).

Taken together these results provide strong evidence that even in the absence of a mature adaptive immune response, L. reuteri can reverse the deficits in (i) oxytocin production in the brain, (ii) social behavior, and (iii) social interaction-induced synaptic transmission. Thus, the prosocial effects mediated by L. reuteri are independent of the adaptive immune system.

## DISCUSSION

Gut microbiota are emerging as a potent modulator brain function and behavior. However, one of the biggest challenges in gut-microbiota-brain axis research remains the identification of the mechanism(s) by which a given bacterial strain regulates a selective behavior or disease state. To understand the precise mechanism(s) through which gut microbes exert their function(s) in the brain can not only help the design of new clinical trials but could also lead to the development of microbe-based, more personalized treatments while minimizing off-target effects.

Our previous work shows that L. reuteri improves social behavior in mouse models for ASD through a mechanism involving biopterin (BH4) signaling in the gut, the vagus nerve, and the oxytocinergic-dopaminergic reward circuitry in the brain (13, 14, 28). Here, using genetic, molecular, behavioral, and electrophysiological approaches, we found that L. reuteri does not require the mature adaptive immune system to improve social behavior in a mouse model for ASD. Accordingly, L. reuteri was able to increase oxytocin levels in the brain and promote synaptic plasticity associated with social reward in the absence of a mature adaptive immune system. These data are unexpected since (i) gut microbes are required for proper development and maturation of the immune system (73); (ii) a majority of immune cells are found in the gut, highlighting the importance of microbe-immune cell interactions (74); and (iii) specifically, L. reuteri facilitates wound healing via the vagus nerve, oxytocin, and the adaptive immune system (36).

While our work shows that the Rag2-mediated mature adaptive immune system is not involved in the L. reuteri-mediated rescue of social deficits, it remains unknown whether other components of the immune system, such as the innate immune response, could be involved. Indeed, gut microbes and microbial products are known to interact with other immune cells types such as macrophages (75, 76), dendritic cells (77, 78), and innate-like T cells such as mucosal associated invariant T cells (79, 80). These cells could act upstream of oxytocin, vagus nerve, or BH4 signaling and play a role in the rescue of social behavior. However, this has proven challenging to study thus far given limitations in the genetic tools available to specifically ablate these cells *in vivo*.

It is also possible that L. reuteri improves social behavior independently of immune cells. For example, L. reuteri, or a metabolite produced by L. reuteri, could interact with epithelial cells in the gastrointestinal tract to either induce production of BH4 or prevent its degradation. Indeed, gut microbes and their metabolites interact with intestinal epithelial cells (81), which express the enzymes required for BH4 synthesis (82). In addition, specific subtypes of epithelial cells, such as enteroendocrine cells, have been shown to stimulate the vagus nerve (83). Interestingly, BH4 has been shown to stimulate the vagus nerve independent of its role as a cofactor for neurotransmitter synthesis (84). Moreover, BH4 has been shown to increase oxytocin release in neurons (85, 86).

In addition, metabolites produced by L. reuteri could directly interact with either enteric nerves or vagal afferent nerves which innervate the intestines (87). For instance, L. reuteri has been shown to produce γ-aminobutyrate (GABA) which is one of the nervous system’s main signaling molecules (88). Future work will aim to understand whether potential interactions between the L. reuteri and/or its metabolites and the innate immune system, intestinal epithelial cells, and/or peripheral nerves affect various phenotypes such as social behavior.

In conclusion, our data presented in this study indicate that L. reuteri improves social behavior independently of the mature adaptive immune system and that the bacteria modulate disparate host phenotypes (wound healing and social behaviors) and organs (skin and brain) through different mechanisms.

## MATERIALS AND METHODS

### Animals.

C57BL/6J (stock no. 000664), *Shank3B*^+/–^ (stock no. 017688) (49), and *Rag2*^−/−^ (stock no. 008449) (52) mice were obtained from Jackson Laboratories (Bar Harbor, ME). *Shank3B^−/−^* mice were generated from *Shank3B^+/–^* × *Shank3B^+/–^* breeding, and littermates were cohoused according to sex. *Shank3B*^−/−^-*Rag2*^−/−^ mice were generated by crossing *Shank3B*^−/−^ mice to *Rag2*^−/−^ mice to yield *Shank3B^+/–^-Rag2^+/–^* mice, which were then crossed together to yield double-knockout mice and WT controls. Littermates were cohoused by sex. All mice were kept on a 12-h light/dark cycle and had access to food and water *ad libitum*. Only male mice were included in the study, since we previously found that female *Shank3B^−/−^* mice display normal social behavior (data not shown). All mice used in these experiments were 7 to 12 weeks of age. Animal care and experimental procedures were approved by Baylor College of Medicine’s Institutional Animal Care and Use Committee in accordance with all guidelines set forth by the U.S. National Institutes of Health.

### Culture and treatment with *L. reuteri*.

*Limosilactobacillus reuteri* 6475 was cultured anaerobically in MRS broth at 37°C in a 90% N_2_/5% CO_2_/5% H_2_ environment as previously described (13, 28). Cultures were centrifuged, washed, resuspended in phosphate-buffered saline (PBS), and frozen at −80°C until use. PBS (vehicle) or L. reuteri (~1 × 10^8^ CFU/mouse/day) was added to the drinking water daily. Mice consumed the treated water *ad libitum* for the duration of the treatment period. Behavioral assays, tissue collection, and electrophysiological recordings were initiated 4 weeks after treatment began.

### Three-chamber test for social behavior.

The three-chamber test for social behavior was assayed on 7- to 10-week-old male mice, as previously described (13, 89). Briefly, mice were first habituated for 10 min an empty 60 × 40 × 23-cm Plexiglass arena divided into three interconnected chambers. Sociability was evaluated during a second 10-min period. The subject could interact either with an empty wire cup (Empty) or a wire cup containing a genotype, age, sex, and treatment-matched stranger conspecific (Mouse). The interaction time was determined by measuring the time the subject mouse spent sniffing or climbing upon either the empty cup or the cup containing the stranger mouse. The empty cup/stranger mouse’s position in the left or right chamber during the sociability period was counterbalanced between trials to avoid bias. The time spent interacting with the empty cup or the mouse was recorded and measured using the automated AnyMaze software by trained, independent observers. The human observer was blind to treatment and genotype during the experiment. Preference for social novelty, which is often measured during the three-chamber test, was not performed since we previously found that *Shank3B^−/−^* mice show a normal preference for social novelty (14).

### Flow cytometry.

Single cell suspensions were prepared using a GentleMACs dissociator (Miltenyi). Spleens were placed whole into C tubes (Miltenyi) containing 3 mL of digestion buffer and RPMI 1640 (Gibco) containing 100 μg/mL DNase I (Sigma) and 500 μg/mL collagenase IV (Sigma). Organs were ground on the dissociator, incubated 15 min at 25°C, ground again, incubated an additional 15 min, and ground one final time. The homogenates were chilled on ice and enzymes deactivated using 10 mM EDTA. The suspensions were filtered through a 40-mm cell strainer for staining, followed by red blood cell (RBC) lysis using eBioscience RBC lysis solution (Thermo Fisher) for 5 min on ice. The homogenates (1/16 of each spleen) were then Fc-blocked using 4 μg/mL anti-CD16/CD32 antibodies (BD Bioscience Biosciences) on ice for 15 min. Antibody staining was performed for 30 min at 4°C. The following fluorescent anti-mouse antibodies were used: CD3 (BV421, dilution 1/100), CD4+FITC (dilution 1/800), IgM (APC, dilution 1/50), and CD45RA (PE, dilution 1/200). DAPI (4′,6′-diamidino-2-phenylindole; 50 μL/3 mL) staining was used to discriminate between living and dead cells.

The flow cytometric data were then analyzed using FlowJo software (BD Bioscience), including the FlowAI plugin. First, FlowAI (2.0) was run to exclude signal acquisition and dynamic range anomalies using the default settings. Second, debris were excluded based on forward scatter (FSC) and side scatter (SSC). Third and fourth, singlets were isolated using FSC-A versus FSC-H, followed by SSC-W versus SSC-H. Fifth, dead cells were exclude based on the DAPI signal. The various immune cell populations were then isolated by using the various immune markers. In particular, the number of mature T lymphocytes was assessed by measuring CD3^+^ CD4^+^ cells and CD3^+^ CD4^–^ cells, and the number of mature B lymphocytes was analyzed based on IgM and CD43RA^+^ expression.

### Immunofluorescence.

Immunofluorescence was performed as we previously described (13). Briefly, mice were deeply anesthetized by inhalation of isoflurane and perfused through the ascending aorta with 10 mL of 0.9% PBS, followed by 30 mL of 4% paraformaldehyde in 0.1 M phosphate buffer (PB). Brains were removed, immersed in the same fixative overnight at 4°C, and subsequently cryoprotected in 30% sucrose (in 0.1 M PB) over 3 days. Coronal sections were cut at 30 μm with a cryostat (Leica Biosystem) and then collected in ice-cold PBS. Slices were rinsed in 0.1 M PB, blocked with 5% normal goat serum plus 0.3% Triton X-100 0.1 M PB (PBTgs) for 1 h of rocking at room temperature, and then incubated for 24 h at 4°C in a mixture of primary antibodies diluted in PBTgs. Sections were then washed (three times with 0.3% Triton X-100 0.1 M PB), incubated in a mixture of secondary antibodies coupled to a fluorochrome, and diluted in PBTgs for 1.5 to 2 h in the dark at room temperature. Sections were rewashed (three times with PBTgs, 0.1 M PB, and 0.05 M PB, respectively, for 5 min each). Slices were mounted onto 2% gelatin-coated slides (Sigma-Aldrich), air-dried, and cover-slipped with a mounting medium (Fluorescence Vectashield H-1200 with DAPI [Vector Labs]). The primary antibodies used were rabbit anti-oxytocin (ImmunoStar, 1:2,000 dilution), while the secondary antibodies were goat anti-rabbit Alexa Fluor 488 (Thermo Fisher Scientific).

Fluorescent imaging and data acquisition were performed on a Zeiss AxioImager Z2 microscope (Carl Zeiss MicroImaging) mounted with an AxioCam digital camera (Carl Zeiss MicroImaging). Images were captured using AxioVision acquisition software (Carl Zeiss MicroImaging). All images within the same set of experiments were acquired at identical exposure times for every channel used to compare fluorescence intensity. Hypothalamic oxytocin-expressing neurons and NeuN-expressing cell numbers were assessed in the well-defined PVN region using the automatic cell counter plugin in ImageJ, as previously described (14), using the following operational sequence: open image file, 16-bit conversion, subtract background, adjust threshold, watershed, and analyze particles. Automatic identification of cell boundaries was validated against the source image. Fluorescence intensity was measured in ImageJ by selecting regions of interest (i.e., oxytocin-positive hypothalamic cell bodies, 30 cells per mouse) using the following operational sequence: open image file, 16-bit conversion, set measurement, ROI manager, and measure. Contrast and brightness were linearly adjusted using Photoshop (Adobe) or ImageJ (NIH) uniformly across all images within the data set.

### Electrophysiology.

Recordings were performed as previously described (13, 90), with minor modifications. Briefly, animals were anesthetized with isoflurane and then decapitated. The brain was rapidly removed from the skull and fixed on a vibroslicer stage (VT 1000S; Leica Microsystems, Buffalo Grove, IL) with cyano-acrylic glue. Acute 220- to 300-μm-thick coronal slices were cut in ice-cold (2 to 3°C) cutting solution containing the following: 87 mM NaCl, 25 mM NaHCO_3_, 25 mM glucose, 75 mM sucrose, 2.5 mM KCl, 1.25 mM NaH_2_PO_4_, 0.5 mM CaCl_2_, and 7 mM MgCl_2_ (equilibrated with a 95% O_2_/5% CO_2_) gas mixture (pH 7.3 to 7.5). Slices were incubated for 20 min at 32°C and then stored at room temperature in a holding bath containing oxygenated standard artificial cerebrospinal fluid (ACSF) containing 125 mM NaCl, 25 mM NaHCO_3_, 25 mM glucose, 2.5 mM KCl, 1.25 mM NaH_2_PO_4_, 2 mM CaCl_2_, and 1 mM MgCl_2_ (equilibrated with 95% O_2_/5% CO_2_) for at least 40 min before being transferred to a recording chamber mounted on the stage of an upright microscope (Examiner D1; Carl Zeiss, Oberkochen, Germany). The slices were perfused with oxygenated ACSF (2 mL/min) containing the GABA_A_ receptor antagonist picrotoxin (100 μM; Sigma-Aldrich, USA) and maintained at 32°C with a Peltier feedback device (TC-324B; Warner Instrument). Whole-cell recordings were performed using conventional patch-clamp techniques. Patch pipettes were pulled from borosilicate glass capillaries (World Precision Instruments, Inc., FL) and filled with the following intracellular solution: 117 mM CsMeSO_3_, 0.4 mM EGTA, 20 mM HEPES, 2.8 mM NaCl, 2.5 mM Mg-ATP, and 0.25 mM Na-GTP. Then, 5 mM Tetraethylammonium Chloride (TEA Cl) was added, the pH was adjusted to 7.3, and the osmolarity was adjusted to 290 mOsm using a Vapro5600 vapor pressure osmometer (ELITechGroup Wescor, South Logan, UT). When filled with the intracellular solution, patch pipettes had a resistance of 2.0 to 3.0 mΩ before seal formation.

Recordings were performed with Multiclamp 700B (Molecular Devices), sampled at 20 kHz with Digidata 1440A (Molecular Devices) interface, filtered online at 3 kHz with a Bessel low-pass filter, and analyzed offline with pClamp10 software (Molecular Devices). The ventral tegmental area (VTA) was visually identified by infrared differential interference contrast video microscopy, and the lateral VTA was determined considering the medial lemniscus and the medial terminal nucleus of the accessory optic tract as anatomical landmarks. Dopaminergic (DA) neurons in this area were identified evaluating the following features: (i) cells firing at a frequency of 1 to 5 Hz and a spike width of >1 ms in a cell-attached configuration, (ii) a membrane capacitance (Cm) of >28 pF, and (iii) the presence of an Ih current and a leak current of >150 pA, when hyperpolarized from −40 mV to −120 in 10-mV steps (91, 92). Passive electrode-cell parameters were monitored throughout the experiments, analyzing passive current relaxations induced by 10-mV hyperpolarizing steps applied at the beginning of every trace. A variation of series resistance (R_s_) of >20% led to the rejection of the experiment. AMPAR/NMDAR ratios were calculated as previously described (13, 90). Briefly, neurons were slowly voltage-clamped at +40 mV until the holding current stabilized (at 200 pA). Monosynaptic excitatory postsynaptic currents (EPSC) were evoked at 0.05 Hz with a bipolar stimulating electrode placed 50 to 150 μm rostral to the lateral VTA. After recording the dual-component EPSC, DL-AP5 (100 μM) was bath applied for 10 min to isolate the AMPAR current, blocking the NMDAR. The NMDAR component was then obtained by offline subtraction of the AMPAR component from the original EPSC. The peak amplitudes of the isolated components were used to calculate the AMPAR/NMDAR ratios.

### Statistical analysis.

Statistical analysis was performed as previously described (14, 28). Data are presented as means ± the standard errors of the mean (SEM). Statistical analyses performed include the unpaired Student *t* test and one- or two-way analysis of variance (ANOVA) with either Tukey’s or Bonferroni test to correct for multiple comparisons as indicated in the figure legends, unless otherwise indicated. *P*, *t*, q, and F values are presented in the figure legends. *P* < 0.05 was considered statistically significant (*, *P* < 0.05; **, *P* < 0.01; ***, *P* < 0.001; ****, *P* < 0.0001). Prism 9 software (GraphPad, La Jolla, CA) was used to perform statistical analyses and generate graphical data representations.

### Data availability.

All data supporting the findings of this study are available within this article and its supplemental material.
