# Correction for Dooling et al., “The Effect of *Limosilactobacillus reuteri* on Social Behavior Is Independent of the Adaptive Immune System”

**DOI:** 10.1128/msystems.00477-23

**Published:** 2023-07-07

**Authors:** Sean W. Dooling, Martina Sgritta, I-Ching Wang, Ana Luiza Rocha Faria Duque, Mauro Costa-Mattioli

Volume 7, No. 6, e00358-22, 2022, https://doi.org/10.1128/msystems.00358-22. Page 9, Acknowledgments paragraph 3: The following should be added as the second sentence: “M.C.-M. has financial interests in Altos Labs, Mikrovia, and Bioavatar, LLC.”

